# Conservative Management of Osteoporotic Vertebral Fractures: A Prospective Study of Thirty Patients

**DOI:** 10.7759/cureus.542

**Published:** 2016-03-24

**Authors:** Siddharth Shah, Arvind B Goregaonkar

**Affiliations:** 1 Department of Orthopaedics, Lokmanya Tilak Municipal Medical College & General Hospital, Sion, Mumbai; 2 Professor and Head, Department of Orthopedics, Lokmanya Tilak Municipal Medical College & General Hospital, Sion, Mumbai

**Keywords:** osteoporotic vertebral fractures, conservative treatment, combined modality treatment, treatment efficacy, osteoporosis

## Abstract

**Introduction:**

Osteoporotic vertebral fractures relate to poorer quality of life and higher long-term mortality. In a resource-poor setup, conservative management assumes great importance as primary means of treatment. We assess the clinico-pathological epidemiology of osteoporotic vertebral fractures and prospectively evaluate the effectiveness of conservative management in their treatment.

**Materials & Methods:**

Thirty consecutive patients, diagnosed with osteoporotic vertebral fracture, underwent the predetermined protocol of conservative treatment (bed rest, titrated analgesia, antiosteoporosis pharmacotherapy, bracing, and supervised physical therapy) after assessment of basic demographic data and clinical examination. They were evaluated every three months for nine months, using visual analog scale (VAS) for backpain, Oswestry Disability Index (ODI), and radiological and hematological parameters.

**Results:**

The mean age of the patients was 66.9 +/-7.6 years and the female: male ratio was 14:1. All (100%) women were postmenopausal and the mean time since menopause was 16.75 +/- 8.12 years. The presenting complaints were backpain (100%) and deformity (53.33%). Neurodeficit was noted in one (3.33%) patient. Higher age was correlated to greater vertebral collapse (p=0.001) and higher kyphotic angle (p=0.002). At nine months after treatment, there was a significant decrease in the VAS score (p<0.0001) and the ODI score (p<0.0001), with the final VAS score improving by 49.25% and the ODI score improving by 47.23% from the baseline. There was progressive increase in vertebral collapse (p=0.0474) with no change in kyphotic or scoliotic angles. With treatment there was a consistent increase in serum calcium (p<0.0001), phosphorous (p<0.0001), and vitamin D3 (p<0.0001) levels, and decrease in parathyroid hormone levels (p<0.0001).

**Conclusions:**

A multidisciplinary conservative treatment is effective as the primary treatment for patients with osteoporotic vertebral fractures. It alleviates pain, decreases disability, reduces morbidity, and is effective in preventing further progression of the disease process clinically, radiologically, and hematologically.

## Introduction

Osteoporotic vertebral fractures are associated with a poorer quality of life and higher long term mortality in patients [1]. The occurrence of one vertebral fracture (even if asymptomatic) quadruples the likelihood of a second fracture, and after a second fracture the risk of further fractures, is 12 times higher [2]. 50% of white women sustain an osteoporotic fracture at some point in their lifetime [3]. Men have a lower but significant osteoporotic fracture risk which peaks 10 years later than in women [3].

Treatment of osteoporotic vertebral fractures is conventionally nonsurgical and conservative. The treatment is primarily aimed at preventing further bone loss, increasing the bone mass, achieving fracture union, and assisting in rapid rehabilitation. Surgical techniques are indicated for chronic mechanical back pain, neurological deficit or spinal deformity. The average in-patient charge for those patients undergoing surgical procedures like kyphoplasty was approximately $17,000 more than that of nonoperative care [4]. The annual cost of treating all osteoporotic fractures in Europe is estimated to be EUR 25 billion [5]. In a resource-limited region, such treatment options place a substantial economic burden on the healthcare setup as well as on the patients, who may forego treatment due to the high cost of the surgical procedures. In such a scenario, a conservative line of management of these fractures assumes great importance as the primary means of treatment.

There is paucity of literature on conservative treatment of osteoporotic vertebral fractures, since it is usually assessed as a control group in the studies on the interventional procedures. Thus, in this study, we aim to assess the clinico-pathological epidemiology of osteoporotic vertebral fractures and prospectively evaluate the effectiveness of conservative management in their treatment.

## Materials and methods

We studied 30 consecutive patients who presented to the outpatient department of a tertiary care hospital between December 2013 and November 2014 and were diagnosed to have an osteoporotic vertebral fracture. Inclusion criteria were acute vertebral fractures presenting with back ache, deformity or neurological deficit, with a densitometric diagnosis of osteoporosis as per the WHO definition [6]. All patients underwent a bone mineral density assessment with dual-energy X-ray absorptiometry (DXA) scan, using the Lunar Prodigy Advance DXA System (GE Healthcare, Madison, WI, USA. Software analysis version 12.30). The exclusion criteria were: pathological fractures secondary to primary or metastatic tumors of the spine, infectious or metabolic bone diseases, previous surgical interventions, severe osteoarthritis of the knees or hips and hemodynamically or medically unfit patients.

The patient assessment included basic history and clinical examination, evaluation of the backpain using visual analog scale (VAS), neurological assessment, and disability assessment using Oswestry Disability Index (ODI). The radiological assessment included fracture location, pattern and grade, with measurement of coronal or sagittal deformity. Serum calcium, serum phosphorus, serum alkaline phosphatase, serum parathyroid hormone and serum vitamin D3 levels were measured and monitored during the course of the treatment using standard assays. The ethical committee approved the plan for the study and a written informed consent was obtained from each patient after proper explanation of the study and treatment protocol.

### Management protocol

All patients eligible for the study, were started on a regimen of conservative treatment, which comprised of bed rest, titrated analgesia, antiosteoporosis pharmacotherapy, brace immobilization, and physical rehabilitation. Bed rest was advised for a short duration only, to overcome the pain due to the acute fracture. Cyclooxygenase inhibitors were primarily used for the relief of the acute pain, in gradually tapering doses, for patients who had no contraindications to their use. Maintenance doses for chronic pain were individualised based on dose titration and side effect profile. Use of opioids (tramadol 25 mg, upto a maximum of 200 mg per day) was restricted to refractory pain nonresponsive to cyclooxygenase inhibitors. The physical rehabilitation was undertaken by a single expert physiotherapist, with a regimen personalised for each patient, as per the Physiotherapy Rehabilitation for Osteoporotic Vertebral Fracture (PROVE) trial methodology [7]. The regimen included manual therapy interventions like low velocity spinal mobilisation, soft tissue mobilisation and postural taping, as well as exercise interventions like active stretches, progressive balance and strength training, and low to moderate intensity weight-bearing aerobic activity [7]. All patients received an antiosteoporosis drug regimen that included bisphosphonates (oral ibandronate 150 mg monthly or oral alendronate 70 mg weekly), oral calcium supplementation (elemental calcium 1000 mg daily), oral vitamin D3 supplementation (60000 IU weekly), and oral vitamin C supplementation (100 mg daily).

All patients were evaluated for their clinical, neurological, radiological, and hematological parameters along with VAS score and ODI score at presentation, at three months, six months, and nine months follow-up. The statistical analysis of the outcomes was done using analysis of variance (repeated measures ANOVA) with a post hoc Tukey test. Variables were corrected for baseline values by subtracting the follow-up measurement from the baseline value, and expressing the difference as a percentage of the baseline value.

## Results

Twenty-eight women (93%) and two men (7%) were enrolled in the study and followed for a period of nine months. The age ranged from 52 to 81 years (mean 66.9 +/- 7.6 years). All women (100%) were postmenopausal, the mean time since menopause being 16.75 +/- 8.12 years. All patients (100%) complained of back pain as the presenting symptom, 53.33% patients also noticed a deformity of the back. One patient (3.33%) presented with neurological deficit at the onset.

The mean visual analog scale (VAS) back pain score at presentation was 6.73 +/- 1.68. There was a significant improvement in VAS score over the time of follow-up (p value <0.0001). There was consistent improvement (mean 20.36%) between each successive trimonthly visit. The final assessment of the VAS score at nine months showed a 49.25% improvement from that at presentation. Forty percent of the patients had an improvement in VAS score by >50%, while 43.33% of the patients had 26-50% improvement at final visit.

Disability as a consequence of the osteoporotic vertebral fracture was measured using the Oswestry Disability Index (ODI). The mean ODI score at presentation was 42.77 +/- 13.77%. Based on the ODI scores, patients were classified into minimal disability (ODI 0-20%), moderate disability (ODI 20-40%), severe disability (ODI 40-60%), crippled (ODI 60-80%), and bedridden (ODI >80%). At presentation, most patients fell in the moderate (40%) and severely (46.67%) disabled groups, while at final assessment most patients fell in the minimal (56.67%) and moderately (36.67%) disabled group. There was a significant improvement in the ODI scores over the time of follow-up (p value <0.0001). There was consistent improvement (mean 22.57%) between each successive trimonthly visit with the maximum improvement noted in the first three months of treatment (28.52% decrease in ODI score). The final assessment of the ODI score at nine months showed a 47.23% improvement from that at presentation. Fifty percent of the patients had 26-50% improvement, while 40% of the patients had >50% improvement in their disability score. The change in the VAS scores for backpain was reflected in the corresponding change in the ODI scores (p value <0.0001).

A total of 693 vertebrae were radiologically evaluated, mean of 23.1 vertebrae per patient. Of these, 45 vertebrae were fractured, with a mean of 1.5 vertebrae involved per patient. Lumbar vertebrae were more commonly involved (23 patients, 76.66%) than dorsal vertebrae (11 patients, 36.67%). Of these, both dorsal & lumbar involvement was seen in six patients (20%). L3 was the most common vertebra involved (22.22%), followed by L1 and L2 vertebrae (20% each). Amongst the dorsal vertebrae, D12 was the most common involved vertebra (17.78%). Forty percent of the patients had Grade I fracture, 23% had Grade II fracture and 37% had Grade III fracture as per Genant’s classification [8]. There was a significant increase in the vertebral height collapse of the involved vertebrae at final follow-up (p value 0.047). No significant change was observed in the kyphotic or scoliotic angles. Increasing age positively correlated with greater vertebral height collapse (p value 0.001) as well as with greater kyphotic angles (p value 0.002). 

Hematological indices showed significant change on conservative treatment with serum calcium (p value <0.0001), phosphorus (p value <0.0001), and vitamin D3 (p value <0.0001) levels showing significant improvement with time. This improvement in the hematological indices was significant between each successive trimonthly follow-up visit. Serum parathyroid hormone levels showed significant decrease over the follow-up period (p value <0.0001), which was maximally noted in the first three months beyond which the levels reached a plateau. No significant changes in the alkaline phosphatase levels were observed.

## Discussion

The study showed a predominantly female population, with the male: female ratio of 1:14. Although European Prospective Osteoporosis Study (EPOS) group had showed almost equal distribution amongst 3174 males and 3614 females, in a ratio of 1 : 1.13 [9], most studies demonstrate a female preponderance of osteoporotic vertebral fractures [10-16]. All women were postmenopausal. Involutional bone loss starts between the ages of 35-40 yrs in both sexes, but there is an acceleration of bone loss in the decade after menopause in the female sex, referred to as type I osteoporosis. Within the first decade after menopause, bone loss affecting the lumbar spine nearly triples in women [17]. Pinheiro MM et al. also stated that menopause is the main factor associated with low trauma fracture [18]. Low calcium and vitamin D intake become additive insults in the microarchitectural deterioration [19].

Back pain was the universal symptom seen in all patients, followed by deformity of the back. Francis RM et al. noted that only one third vertebral fractures come to medical attention where they typically present with acute back pain, but other presentations include deformity secondary to loss of height and increasing kyphosis [20]. Lyritis et al. studied the natural history of osteoporotic vertebral fractures in 210 postmenopausal women and identified two groups [21]. In individuals with type I fractures, the osteoporotic vertebral fractures were radiographically evident and a single episode of pain was severe and acute, persisting for four to eight weeks. In type II fractures, the fracture was not clear radiographically, but a wedge deformity gradually developed over the next few months. The pain in type II fractures was less severe and of shorter duration than type I, but a new attack of pain occurred after six to 16 weeks and often recurred over a period of six to 18 months. Neurological involvement as a direct consequence of the vertebral fracture was seen only in one patient. Spinal cord compression and myelopathy is not a common finding in vertebral osteoporotic fractures [22-23], as there is no structural interruption of the posterior wall or middle column of the vertebral body. Although rare, some patients with osteoporotic vertebral compression fractures may present with neurological involvement [24].

Conservative treatment is the traditional line of management of osteoporotic vertebral fractures [20]. Short period of bed rest, analgesic medications, antiosteoporosis pharmacotherapy, bracing support for the fracture along with guided physical therapy and postural correction aid in lasting alleviation of the pain. According to Park YS et al., patients with established osteoporotic fractures should be confined to bed for two to three days, accompanied by the use of analgesics, hot packs, massage, and lumbar orthosis [24]. Francis RM et al. stated that the management of patients with acute vertebral fractures should include measures to reduce pain, improve mobility, and treatment for osteoporosis [20]. For the prevention and treatment of chronic pain, the back muscles should be strengthened with manual therapies and exercise intervention. Physical rehabilitation has a beneficial effect on bone metabolism, bone turnover, and bone mineral content. A number of systematic reviews and meta-analyses report the positive effects of exercise on bone mineral density, muscle strength, and quality of life, in men and women with osteoporosis or low BMD [7]. The use of a walking assistance device or orthosis can also help prevent patients with osteoporosis from sustaining a fall injury [24]. Spinal orthoses reduce pain by reducing mobility, decreasing postural flexion and providing axial support in patients with muscle fatigue and spasm. 

Complications associated with conservative management should be borne in mind while instituting the treatment. Bed rest causes accelerated bone loss [25] and loss of muscle strength [11]. Immobilization also leads to adverse effects on cardiac and pulmonary function in the geriatric population. Marshall D et al. advocated against the use of brace or corset as it immobilizes the spine, thereby aggravating bone loss and wasting of the surrounding muscles [26]. Cyclooxygenase inhibitors are associated with gastrointestinal side effects like ulcers and gastritis, while the opioids can cause sedation, constipation, and urinary retention. Use of bisphosphonates can lead to oesophageal irritation, osteonecrosis of jaw and atypical femoral fractures.

With conservative treatment, there was a significant improvement in the VAS score for the back pain. Pain alleviation resulted in a concomitant decrease in the disability arising out of these fractures, thereby attaining better functional outcomes. Diamond HT et al. [13], had noted 61% reduction in VAS pain scores at six weeks following initiation of conservative treatment and 71% reduction at six to 12 months. They also noted 31% improvement in physical functioning measured by Barthel index, at six weeks, which increased to 39% improvement at six to 12 months. The patients with <25% improvement in pain score may indicate the transition to chronic back pain resulting from secondary alterations in the mechanics of vertebral articulation, anterior wedge collapse, paraspinal muscle fatigue, and subluxation of the arthritic facet joints.

Deformity of the back was the second most common complaint at presentation. According to Old JL et al., as a consequence of osteoporosis, trivial applied force causes the anterior part of the body to crush, forming an anterior wedge fracture, and over time these collapsed anterior vertebrae fuse together to form a kyphotic deformity [14]. Older age and low bone mineral density would be predictors of greater vertebral collapse and kyphoscoliotic deformity, reiterating the findings noted by Francis et al., who stated that vertebral deformities involving the loss of mid-vertebral height (wedging and compression) were significantly related to low bone mineral density [20].

Compression fractures can occur anywhere from occiput to sacrum, although they usually occur at the lumbo-dorsal junction, namely, T8 to L1 and L4 vertebrae, secondary to higher weight bearing loads and dynamic forces acting at these levels [14,27]. Twenty to thirty percent vertebral compression fractures are multiple [14]. Isolated osteoporotic fractures above the level of T4 are uncommon and, even in patients with osteoporosis, should raise the suspicion of another underlying disease process [12]. In the study, lumbar vertebrae were most commonly involved, with L3 vertebra being the most common. Over time, multiple osteoporotic fractures accumulate, resulting in significant loss of spinal height [14]. During the course of the treatment, there was progressive increase in the vertebral body collapse. The height loss was maximally observed in the initial three months, beyond which it reached a plateau as a consequence of fracture union by collapse, effective bracing, back strengthening physiotherapy, and increased load bearing capacity of the vertebral bodies.

The serum calcium, phosphorus, and vitamin D3 values showed improvement with conservative treatment, a direct result of the supplementation of calcium, vitamin D3, and bisphosphonates. Vitamin D plays a key role in calcium-phosphorus homeostasis. When vitamin D deficiency is not an issue, upto 30% to 40% of total dietary calcium is absorbed [24]. Thus supplementation of calcium and vitamin D synergistically improve bone mineralisation. Calcium monotherapy is not recommended for patients with severe osteoporosis with vertebral compression fractures; however, concomitant antiosteoporosis drugs are essential [24]. Vitamin D has also been known to play a key role in increasing muscle strength, improving balance, and reducing the occurrence of fall and injuries [28]

Serum parathyroid hormone values showed decline over the course of the treatment, without a significant change in the alkaline phosphatase levels. Diamond HT et al. [13], had noted the finding of vitamin D deficiency in 79% of the subjects, associated with secondary hyperparathyroidism in 54% of the subjects in the same cohort. The calcium-PTH-vitamin D negative feedback cycle is active in maintaining the calcium-phosphorus balance in the body. Calcium and phosphorus replenishment abolishes the feedback secondary hyperparathyroidism leading to fall in the serum PTH levels. 

The drawbacks of our study are a small sample size of patients, lack of a comparative control group and a short period of observational follow-up. The conservative management protocol outlined in our study can be declared to be effective only if it is compared with a placebo treatment. However, there are no such trials reported in literature.

## Conclusions

Conservative treatment effectively treats the pain and disability caused by osteoporotic vertebral fractures. The clinical improvement is simultaneously accompanied by radiological and hematological improvement, preventing further progression of the disease process. The benefits of treatment are consistent and sustained over a significant period of time. The treatment regimen has to be individualized to meet the needs of each patient using a multidisciplinary approach. When followed in a systematic and planned manner, it is effective in hastening the recovery from pain, improving mobility and flexibility, alleviating the disability, and restoring independence to the lifestyle of the patient. Thus, conservative treatment is advocated as the primary means of treatment for patients with osteoporotic vertebral fractures.
